# Integrated postpartum family planning utilization with maternal health services and associated factors in governmental hospitals of northeast Ethiopia: institution-based cross-sectional study

**DOI:** 10.11604/pamj.2026.54.11.50760

**Published:** 2026-05-15

**Authors:** Delelegn Tsegaye Zikie, Misra Abdulahi Ahmed, Gurmesa Tura Debelew

**Affiliations:** 1 Department of Population and Family Health, Faculty of Public Health, Institute of Health, Jimma University, Jimma, Ethiopia,; 2 Department of Midwifery, College of Medicine and Health Sciences, Wollo University, Dessie, Ethiopia

**Keywords:** Postpartum family planning, service integration, maternal health service, Ethiopia

## Abstract

**Introduction:**

postpartum family planning (PPFP) is one of the least utilized reproductive health services in Ethiopia. Its integration with maternal health services helps to improve postpartum contraceptive use. Although integrating PPFP with maternal health services can improve postpartum contraceptive uptake, implementation of integrated services remains suboptimal in many settings. The study assessed utilization of integrated PPFP and associated factors in government hospitals of northeast Ethiopia.

**Methods:**

a facility-based cross-sectional study design was employed. The study was conducted in four hospitals with a total of 607 participants. Data were collected using face-to-face, interviewer-administered standardized questionnaires and analyzed with SPSS version 26. Variables with a p-value less than 0.25 in the bivariable analysis were included in the multivariable logistic regression. Variables with p-values less than 0.05 at 95% CI in the multivariable analysis were considered statistically significant.

**Results:**

the level of integrated postpartum family planning utilization with maternal health services was 25% (95% CI: 21.6% - 28.5%). Factors associated with utilization were hospital level (AOR = 0.42; 95% CI: 0.20 - 0.80), previous contraceptive use (AOR = 0.40; 95% CI: 0.20 - 0.80), currently pregnant (AOR = 6.80; 95% CI: 1.50 - 29.50), received family planning counseling (AOR = 0.17; 95% CI: 0.10 -0.30), service provider support (AOR = 3.60; 95% CI: 1.80 - 7.00) and women’s decision on contraceptive use during the same-day hospital visit (AOR = 3.60; 95% CI: 1.80 - 7.00).

**Conclusion:**

utilization of the integrated PPFP service was low. Strengthening PPFP integration across all hospital levels through provider training and routine counselling is recommended.

## Introduction

Postpartum family planning (PPFP) integration with maternal and child health services is the provision of family planning alongside maternal and child health services during the same health facility visit, within the same facility, and by the same provider to improve the use of modern contraceptive method [1]. Postpartum family planning integration with maternal and child health (MCH) services is a critical opportunity to address contraceptive needs during the postpartum period. Effective integration gives benefits for improved reproductive health outcomes. Conversely, low PPFP integration constitutes a missed opportunity within maternal and child health programs [2]. Health care providers attempted to integrate maternal health services; however, inadequate training and limited time posed major barriers. Following integration, increases were observed in knowledge, intentions to use contraception, and the number of new family planning acceptors [3]. The integration of PPFP with maternal and child health services varied across health facilities, ranging from 5.3% to 63%. The proportion of clients receiving maternal and child health services increased when family planning services were integrated. Distance to the health facility was identified as a factor influencing PPFP integration [4].

A study conducted in India showed that providing family planning information during antenatal care in the third trimester, delivery, and the postpartum period was positively associated with the use of modern contraceptives in the postpartum period [5]. Integrating FP with MCH services benefits the health system, women, and families; however, it also presents challenges due to fragmented MCH services across different units within health facilities. Effective PPFP integration with other MCH services requires additional training for health care providers and revisions to existing training guidelines to enhance service quality [4]. Effective PPFP service integration requires active involvement of policymakers guided by evidence, as it aligns with the priorities of the Sustainable Development Goals. In the Sidama region of Ethiopia, the unmet need for contraception was 30%, showing a substantial gap in PPFP utilization [6]. In sub-Saharan Africa, less than half of women gave birth at a health facility with a skilled attendant. Reaching women who are living in remote areas requires outreach services by health care providers. Collaboration with multiple sectors and the involvement of supporting organizations help to address the challenges associated with PPFP service integration [1].

Service integration provides time efficiency, convenience, and better utilization of health services. However, its implementation is hindered by limited human resources, logistical challenges, weak community linkages, sociocultural barriers, and variability in health service organization and professional commitment. Despite these challenges, integration remains feasible and beneficial for both clients and healthcare providers [7]. Postpartum women visit health facilities to seek various health services for themselves or their children [8]. This presents an opportunity to provide various MCH services, including PPFP. In Ethiopia, the unmet need for postpartum family planning was 78% [9]. Evidence indicates that family planning counselling during immunization services is more frequently provided at health posts than at hospitals, highlighting missed opportunities for service integration at the hospital level [10,11]. Therefore, this study aimed to assess the utilization of integrated PPFP service and associated factors at government hospitals in northeast Ethiopia.

## Methods

**Study design:** an institution-based cross-sectional study design was conducted.

**Study setting and period:** the study was conducted in the government Hospitals of northeast Ethiopia. These are Dessie Referral Hospital, Debre Berhan Referral Hospital, Kemise General Hospital, and Woldia General Hospital. All hospitals provide maternal and child health services. These hospitals were randomly selected from the four zones of northeast Ethiopia, specifically the Amhara Region, with one hospital chosen from each zone. The zones represented were the South Wollo zone, North Wollo zone, Oromia special zone, and North Shoa zone. The selected hospitals include both referral and district hospitals located in northeastern Ethiopia. These hospitals serve a large number of clients from the surrounding areas. The study period was from March to June 30, 2023.

**Source population:** all postpartum women who had given birth within the past year in northeast Ethiopia.

**Study population:** postpartum women who attended maternal and child health (MCH) services at selected hospitals in northeast Ethiopia.

**Inclusion criteria:** postpartum women who utilized maternal and child health services in hospitals and who had given birth within one year before the survey.

**Exclusion criteria:** postpartum women who were seriously ill or had a mental illness, and women who experienced obstetric complications during their hospital visit.

**Sample size determination:** this study is nested within a larger interventional project. The sample size was computed using G*Power 3.1.9.7 statistical software. Considering the proportion of women who received integrated PPFP services as 12% (based on a study conducted in the Somali region, Ethiopia [12], with an expected effect size of 8.5%, 80% power, 5% level of significance, and a 10% non-response rate. The final sample size for this study was 607 participants.

**Sampling technique:** a simple random sampling technique was used to select study participants. Lists of eligible participants were prepared using the clients´ MCH register or registration book from each MCH unit. Participants were then selected randomly using the lottery method, with proportional allocation applied to each MCH unit. The number of participants from each hospital was determined using proportional allocation to size based on the average client flow taken during the three months preceding the survey.

**Data collection tool:** a structured, pre-tested, face-to-face interviewer-administered a standardized questionnaire that was prepared by adapting from existing literature and tailored to the local context and purpose of the study [5,13]. The questionnaire was developed in English and translated into the local language (Amharic) by a language expert to ensure comprehension by both data collectors and study participants.

**Data collection:** four supervisors, each holding a Master´s degree in Public Health (one per hospital), and eight data collectors with Bachelor of Science degrees in Midwifery (two per hospital) were recruited. Data collectors and supervisors received three days of training covering data collection procedures, the importance of time management, steps to follow before, during, and after data collection, and verification of collected data. These procedures were strictly followed throughout the data collection period. Data collectors and supervisors first introduced themselves to participants before conducting the interviews. Collected data were verified daily by supervisors before the start of the next day´s data collection. Any issues that arose during data collection were addressed in consultation with the research team. Data were collected by healthcare providers who were not employed at the health facilities where the study was conducted.

**Dependent variable:** integrated postpartum family planning utilization.

**Independent variables:** sociodemographic factors: age, religion, distance to hospital, residence, marital status, educational status, and waiting time at hospital. Obstetric factors: gravidity, parity, previous ANC visits, place of delivery, number of children, and previous use of contraceptive methods. Client-centered counseling: that prioritizes the needs, preferences, and circumstances of the individual woman, rather than focusing on provider goals [14]. Hospital level: referral hospital, district hospital.

**Operational definitions:** postpartum family planning service integration is the provision of family planning counseling services and/or contraceptive methods to postpartum women attending hospitals for delivery, postnatal care (PNC), child immunization, child care, or prevention of mother-to-child transmission (PMTCT) services [15].

Integrated PPFP utilization (level 1 PPFP integration) refers to postpartum women who received family planning information and communication materials, counseling, and referral services, in addition to being offered at least one short- or long-acting contraceptive method [10]. Non-use of integrated PPFP services (Level 0 PPFP integration) refers to postpartum women who received neither family planning counseling nor any contraceptive method during their hospital visit. Maternal and child health services: health care services delivered through the delivery unit, postnatal care, child immunization, PMTCT unit, and under-five outpatient clinics. Currently pregnant: in this study, it refers to women who come to attend for ANC and are given postpartum family planning counseling during this visit. Unmet need for postpartum family planning: women who wish to use a contraceptive method to space or limit the number of children but are unable to obtain a contraceptive method [8].

**Data processing and analysis:** collected data were cross-checked, cleaned, coded, and entered into EpiData 3.0 software before being exported to SPSS version 26 for statistical analysis. Both descriptive and analytical statistical methods were employed. Descriptive statistics, including percentages, frequencies, means, and standard deviations, were used to present demographic and key study variables. Data were displayed using tables, bar graphs, and pie charts. All independent variables that showed an association in the bivariate logistic regression analysis with a p-value less than 0.25 were entered into the multivariable logistic regression model. Multicollinearity was assessed. The Hosmer-Lemeshow goodness-of-fit test indicated that the model adequately fit the data (p = 0.70). Crude and adjusted odds ratios with 95% confidence intervals were computed, and variables with p-values less than 0.05 in the multivariable logistic regression model were considered significantly associated with the use of integrated PPFP.

**Ethical considerations:** this study was approved by Wollo University, College of Medicine and Health Sciences, School of Nursing and Midwifery Ethical Review Committee (Ref. No. SNM/355/22) and Jimma University Institute of Health Institutional Review Board. Written informed consent was obtained from each participant, and no minors under 18 years of age were involved. The study was conducted in accordance with the principles of the Declaration of Helsinki. All participants were informed about the aim of the study and its potential contribution to improvements in maternal and child health services. Participants who were unwilling to participate were informed of their right to refuse or withdraw at any time during the interview. All data obtained from participants were kept anonymous and confidential.

## Results

**Socio-demographic characteristics of participants:** a total of 607 study participants were involved. The majority of postpartum women, 464 (76.4%), were aged 18-30 years, with a median age of 28 years. More than half of the participants, 330 (54.4%), were followers of the Orthodox religion. Most participants, 467 (77%), intended to have four or more children. About 129 (21.2%) of the participants already had four or more living children (Table 1).

**Table 1:** sociodemographic characteristics of respondents on postpartum family planning integration with selected maternal health services in northeast Ethiopia, 2023/24 (n=607)

Variables category		Frequency	Percentage
Age (years)	18-30	464	76.4
	31-50	143	23.6
Religion	Orthodox	330	54.4
	Muslim	258	42.6
	Protestant	12	2
	Catholic	7	1
Marital status	Married	573	94.4
	Divorced	23	3.8
	Widowed	3	0.5
	Single	8	1.3
Educational status	Cannot read & write	133	22
	Primary	252	41.5
	Secondary	129	21.2
	College & above	93	15.3
Time to reach the hospital	< 1 hour	427	70.3
	>1 hour	180	29.7
Planned number of children	1-3 children	140	23
	>4 children	467	77
Number of living children	1	204	33.7
	2	162	26.7
	3	112	18.4
	4	78	12.8
	>5	51	8.4
Time of last delivery	< 3 months	354	58.4
	3-5 months	80	13.1
	6-12 months	173	28.5

**Unmet need for postpartum family planning use:** more than half of participants, 364 (60%), experienced unmet need for PPFP.

**Postpartum family planning integration with maternal and child health services:** only a small proportion of women, 16 (8%), visited referral hospitals specifically to access postpartum family planning (PPFP) services. At higher-level hospitals, PPFP is often overlooked and receives limited emphasis as part of preventive health care services. The majority of participants visited health facilities for maternal and child health services. Almost one-fourth of the participants, 142 (23%), were delivered at the selected hospitals, which provide a good opportunity to offer postpartum family planning counseling and services during delivery. Only 152 (25%) postpartum women received PPFP services, whereas 271 (44.6%) of participants did not receive any additional maternal and child health services despite visiting hospitals for various maternal and child health services. Similarly, the majority of participants, 308 (77%), did not receive integrated postpartum family planning along with other maternal and child health services at referral hospitals. The level of utilization of integrated PPFP services with maternal and child health services was 152 (25%) (95% CI: 21.59 - 28.49%).

**Factors associated with postpartum family planning integration:** in the bivariable analysis, variables such as age, time to reach hospitals, planned number of children, and reason for hospital visit had a p-value less than 0.25 and were therefore included in the multivariable logistic regression analysis. Several factors were found to be significantly associated with the use of integrated postpartum family planning services. Postpartum women at referral hospitals had 58% lower odds of utilizing integrated PPFP services than those at general hospitals (AOR = 0.42; 95% CI: 0.20 - 0.80). Postpartum women with no prior family planning experience were less likely to receive integrated PPFP services compared to those who had previously used family planning (AOR = 0.40; 95% CI: 0.20 - 0.80). Postpartum women who accessed maternal and child health services based on the healthcare provider´s recommendation were more likely to receive integrated PPFP services compared to those who did not receive such support (AOR = 3.60; 95% CI: 1.80 - 7.00). Participants who did not discuss PPFP with a healthcare provider were less likely to receive integrated PPFP services compared to those who did (AOR = 0.17; 95% CI: 0.10 - 0.30). Women who reported difficulty making a same-day decision about contraceptive use were less likely to receive integrated PPFP services than their counterparts (AOR = 0.26; 95% CI: 0.10 - 0.50). Women who were not currently pregnant had higher odds of receiving integrated family planning counseling services than those who were pregnant (AOR = 6.80; 95% CI: 1.50 - 29.50) (Table 2).

**Table 2:** bivariable and multivariable logistic regression on the factors associated with postpartum family planning integration with other maternal health services in northeast Ethiopia, 2023/24 (n=607)

Variables	Integrated PPFP Utilization		COR (P-value)	AOR (95% CI)	P-value
	Yes n (%)	No n (%)			
**Hospital level**					
Referral hospital	92(23)	308(77)	0.73(0.10)	0.42(0.20-0.80)	0.01*
General hospital	60(29)	147(71)	1	1	
**Currently pregnant**					
No	149(26.1)	422(73.9)	3.88(0.02)	6.8(1.50-29.50)	0.011*
Yes	3(8.3)	33(91.7)	1	1	
**Previous FP use**					
No	73(20.1)	291(79.9)	0.50(0.001)	0.40(0.20-0.80)	0.006*
Yes	79(32.5)	164(67.5)	1	1	
**Reason for hospital visit**					
For other MCH services	124(23.6)	402(76.4)	0.58(0.035)	1.40(0.60-3.00)	0.40
For FP service	28(34.6)	53(65.4)	1	1	
**Service provider support**					
Got service provider support	78(51.7)	73(48.3)	3.20(0.000)	3.60(1.80-70)	0.001*
No, got service provider support	72(39.1)	112(60.9)	1	1	
**Received PPFP counseling**					
No	39(10.7)	327(89.3)	0.13(0.000)	0.17(0.10-0.30)	0.001*
Yes	113(46.9)	128(53.1)	1	1	
**Age (years)**					
15 -30	117(27)	317(73)	1.45(0.085)	1.40(0.70-3.00)	0.30
31-49	35(20.2)	138(79.8)	1	1	
**Time to reach the hospital**					
< 1 hour	120(28.1)	307(71.9)	1.80(0.008)	1.60(0.80-3.00)	0.14
1 hour and above	32(17.8)	148(82.2)	1	1	
**Planned number of children**					
1-3	29(20.7)	111(79.3)	0.73(0.17)	0.60(0.30-1.20)	0.10
4 and above	123(26.3)	344(73.7)	1	1	
**Women’s decision on contraceptive use during the same-day hospital visit**					
No	18(7.5)	222(92.5)	0.14(0.000)	0.26(0.10-0.50)	0.001*
Yes	134(36.5)	233(63.5)	1	1	

FP: family planning, MCH: maternal and child health, PPFP: postpartum family planning, COR: crude odds ratio, AOR: adjusted odds ratio, *: statistically significant at p < 0.05

## Discussion

This study aimed to assess the utilization of integrated postpartum family planning (PPFP) with maternal and child health (MCH) services, as well as the associated factors, among postpartum women in northeast Ethiopia. The overall utilization of integrated PPFP with MCH services was 25% (95% CI: 21.59-28.49%), which is low compared to a previous study conducted in Ethiopia (72.9%) [6]. In India, the level of MCH-FP integration across facilities ranged from 5.3% to 63.0%, while in Jharkhand, PNC-FP integration was 60% [11]. Knowledge on family planning integration with other maternal health services was 65.9%, and half of the participants (53.5%) used PPFP in Ethiopia [11,16].

The reason for this discrepancy might be due to differences in the socio-demographic characteristics of respondents. The study area included both urban and rural populations, who may have limited awareness of the availability of integrated services. Variations in the study period also contributed to these differences. Differences in study design and populations might also have their own effect. In this study, participants were postpartum mothers who visited various MCH services and had given birth, whereas other studies included different study populations. Furthermore, the focus of this study was on PPFP service integration, while others were not.

The level of PPFP integration utilization with other MCH services was in line with a study in Bihar, where 28.1 to 35.2% of clients who received ANC service also received FP counseling [5,9]. However, fewer than 10.9% of clients received PNC, and only 2.2 - 4.1% of clients who received child health services also received FP services [17]. The other reasons for poor integration of PPFP might be due to inadequate monitoring and evaluation by healthcare managers, high caseload by healthcare providers, limited attention to FP services in referral hospitals, and insufficient PPFP training for service providers.

However, the use of integrated PPFP with MCH was higher than in studies conducted in Burundi (20%) [18], Kenya (5.5%) [11], India (16.3%) [19], and Kebribeyah Town, Ethiopia 12.3% [12]. Utilization of family planning immediately after birth in Addis Ababa was 12.9% [20]. This difference may be attributed to low awareness of the benefits of immediate postpartum contraceptive use and the perception that fertility does not return even if a woman is not breastfeeding. About 92 (23%) of postpartum women at referral hospitals received PPFP service, but 169 (42.3%) of women didn´t receive additional MCH services even if they came to hospitals for various reasons. In India, the use of MCH-FP integrated services ranged from 16.3% to 63%, Kenya (5.5%) of clients used MCH-FP integration compared with 14.8% in Bondo Hospital [11,18].

At Bondo Hospital, 20.2% of clients who accessed child health services also received family planning [11]. This finding was similar to that of the present study. The reasons for this might include the workload of healthcare providers, inadequate monitoring, and poor mentoring. Differences in the socio-demographic characteristics of respondents, study design, and types of data used (the previous study used secondary data) may also contribute. Despite a high proportion of women visiting health facilities for MCH services, only a small proportion received family planning information [5,21]. This finding was similar to that of the present study, in which many mothers visited for delivery, PNC, child immunization, under-five child care, and PMTCT services, but most missed the opportunity for family planning counseling. Due to low PPFP service coverage, the unmet need for PPFP was 22% [22,2,3].

In this study, the factors associated with the use of integrated PPFP within MCH services included hospital level, previous FP use, family planning counseling, healthcare provider support, currently pregnant, and women´s decision to initiate contraceptive use on the same day of hospital visit. Postpartum women seeking maternal and child health services at referral hospitals were less likely to receive integrated postpartum family planning services compared to those attending district hospitals. This finding is plausible because referral hospitals typically face high patient volumes, increased workloads among health care providers, and a greater burden of complicated cases, which may lead to postpartum family planning services receiving lower priority. Furthermore, hospital managers at higher-level facilities may focus more on curative care than on preventive services. Other contributing factors may include a lack of healthcare provider commitment, inadequate monitoring, and limited internal mentoring activities. This finding highlights the need for program and regional health managers to focus on comprehensive maternal health care, ensuring that every postpartum woman seeking maternal and child health services at any facility level can access her preferred family planning service.

Pregnant women were less likely to receive family planning counseling services than non-pregnant women among those who visited hospitals for maternal health services. This finding indicates that the maternal continuum of care is not well organized. Family planning counseling should be initiated during antenatal care visits at health facilities to help women prepare to use their preferred contraceptive method after childbirth. This finding implies that health care program managers and health care providers should strengthen family planning counseling, particularly during pregnancy, since more women attend health facilities for ANC services.

In this study, women who independently decided to use contraception on the same day were more likely to receive integrated PPFP services. This finding is supported by a study conducted in Tanzania [24]. Clients were more likely to receive a modern contraceptive method when they had information on two or more methods, discussed family planning with their partner, made their own FP decisions, and received FP information from multiple sources [25]. This finding implies that women´s empowerment and autonomous decision-making are crucial for postpartum family planning services. This improves modern contraceptive use and a reproductive rights-based approach as supported by evidence [26].

In this study, previous contraceptive use was associated with the receipt of integrated PPFP services within MCH units. Prior family planning use can influence how women engage with available integrated services. These factors may affect a woman´s likelihood of requesting, accepting, or being offered FP services during MCH visits. A postpartum woman who has previously used contraception might be aware of FP services, more confident discussing them, and more likely to request such services during MCH visits. This can prompt providers to deliver integrated FP services, effectively activating integration that already exists within the system. Conversely, women with no prior FP use may be less likely to initiate such discussions, leading to missed opportunities for integrated care even in well-prepared facilities. Thus, individual factors can influence whether system-level integration translates into actual utilization. This finding was supported by a study done in Hawassa, Ethiopia [27]. Women who were not currently pregnant had higher odds of receiving integrated PPFP services compared to those who were pregnant. This suggests that healthcare providers in antenatal care units may not adequately counsel pregnant women on family planning options, including readiness and the appropriate timing to start contraceptive use. This finding is supported by previous studies, which emphasize that PPFP counseling should begin during pregnancy [23]. It highlights the need for program managers to integrate family planning counseling into antenatal care services.

This study has several implications for healthcare providers, administrators, researchers, and policymakers. The findings highlight the need for all stakeholders involved in maternal and child health care to commit to improving outcomes by preventing unintended pregnancies during the postpartum period. Given the persistently high unmet need for PPFP in Ethiopia, integrating PPFP with MCH services is a crucial strategy to address this challenge [15,28]. In many hospitals, PPFP often receives little attention and is perceived as a routine service to be provided only by lower-level facilities, such as health centers and health posts. This study also highlights the importance of providing client-centered counseling to all postpartum women across all MCH units within hospital settings [29].

**Strength of the study:** this study used a large sample size and involved different hospital levels in four zones of northeast Ethiopia. Study participants were also involved from all MCH units. Being a baseline study gives baseline data for researchers and helps to design interventions based on the gaps identified.

**Limitations:** cross-sectional design limits the ability to establish cause-and-effect relationships, and the findings may not be generalizable to all postpartum women in northeast Ethiopia. The study primarily focused on individual-level factors and did not adequately address supply-side factors, such as health system structures and healthcare provider practices. The analysis of individual-level factors can identify associations, but it cannot determine causal relationships.

## Conclusion

Postpartum family planning service integration with MCH services in government hospitals of northeast Ethiopia was low. Hospital type, previous FP use, receipt of counselling, healthcare provider support, currently pregnant, pregnancy status, and women's immediate decision-making were significantly associated with receipt of integrated PPFP services. Strengthening provider capacity and routine PPFP counselling across all MCH units is essential.

### What is known about this topic

- The magnitude of postpartum family planning utilization has been examined;
- Factors associated with postpartum family planning utilization have been identified in community-based studies;
- Health system factors influencing the integration of family planning services have also been explored.

### What this study adds

- We found that the level of integrated postpartum family planning use with maternal and child health services in government hospitals was 25%, which is low;
- Our study identified six key factors significantly associated with utilization of integrated PPFP services: hospital level, previous contraceptive use, current pregnancy, receipt of family planning counseling, provider support, and women’s decision-making regarding contraceptive use during the same-day hospital visit;
- We identified 60% of postpartum women had an unmet need for PPFP at maternal and child health units.
